# Comprehensive Analysis of *IDD* Transcription Factors and Their Expression Profiling Under Pathogen Stress in Wheat

**DOI:** 10.3390/biology15120904

**Published:** 2026-06-09

**Authors:** Yanzhen Wang, Shikai Lyu, Yanqi Wang, Jialu Li, Xia Liu, Menglin Lei

**Affiliations:** 1 Center for Agricultural Genetic Resources Research, Shanxi Agricultural University, Taiyuan 030031, China; 2 Academy of Agricultural and Forestry Sciences, Qinghai University, Xining 810016, China; 3 College of Agriculture, Shanxi Agricultural University (Institute of Crop Sciences), Taigu 030801, China

**Keywords:** *Triticum aestivum* L., IDD transcription factor, expression pattern, disease resistance, abiotic stress

## Abstract

INDETERMINATE DOMAIN (IDD) transcription factors are plant-specific regulators essential for plant development and stress adaptation. As a globally important staple crop, common wheat (*Triticum aestivum* L.) is frequently threatened by fungal diseases such as powdery mildew and stripe rust. To date, however, the *IDD* gene family in wheat has not been systematically characterized, and its roles in biotic stress responses remain unclear. In this study, we performed genome-wide identification and a comprehensive analysis of the *TaIDD* gene family. A total of 41 *TaIDD* genes were identified, which were unevenly distributed across 15 chromosomes and divided into four phylogenetic groups. Synteny and selective pressure analyses demonstrated that segmental duplication was the main driver of family expansion and that *TaIDD* genes underwent strong purifying selection during evolution. *Cis*-acting element analysis revealed abundant hormone- and stress-related elements in their promoter regions. Transcriptome and RT-qPCR analyses indicated that *TaIDD* genes exhibited distinct expression patterns under abiotic and biotic stress. Notably, *TaIDD13, TaIDD19, TaIDD27, TaIDD37*, *TaIDD39*, and *TaIDD41* were significantly induced by multiple fungal pathogens, suggesting their potential involvement in stress-responsive pathways that may be related to disease resistance. Subcellular localization analysis further confirmed that TaIDD39 was exclusively localized in the nucleus, consistent with its function as a transcriptional regulator. Our findings provide insights into the evolutionary characteristics and stress-response mechanisms of *TaIDD* genes and highlight *TaIDD39* and other potential candidates that may serve as valuable resources for wheat molecular breeding to enhance broad-spectrum disease resistance and stress tolerance.

## Simple Summary

In this study, we systematically identified 41 *TaIDD* genes in wheat and analyzed their evolutionary relationships, chromosomal distribution, and regulatory elements. Through the mining of public transcriptome datasets and experimental validation, we found that several genes, particularly *TaIDD13*, *TaIDD19*, *TaIDD27*, *TaIDD37*, *TaIDD39*, and *TaIDD41*, exhibited elevated expression under drought, heat, *Blumeria graminis* f. sp. *tritici* (*Bgt*), and *Puccinia striiformis* f. sp. *tritici* (*Pst*) stresses. Overall, this work provides a valuable genetic resource and highlights promising candidate genes for future research aimed at improving wheat stress tolerance and disease resistance.

## 1. Introduction

The INDETERMINATE DOMAIN (IDD) proteins constitute a family of plant-specific transcription factors characterized by a conserved IDD DNA-binding domain and serve as pivotal regulators of plant growth, development, and environmental stress responses across multiple species [1,2]. Beyond governing core developmental processes—including root system architecture formation, shoot meristem maintenance, leaf morphogenesis, and flowering transition [3]—*IDD* transcription factors are increasingly implicated in complex phytohormone signaling cascades and stress response networks [4,5]. These proteins appear to bridge growth plasticity and stress tolerance, linking plant development with adaptation to adverse abiotic and biotic cues. Their versatile regulatory roles in fundamental physiological processes render them promising targets for crop molecular breeding, offering substantial potential to improve yield, quality, and stress resilience [6].

In recent years, accumulating evidence has unraveled the precise molecular mechanisms and regulatory pathways underlying *IDD* gene function, although in-depth functional investigations remain largely limited to model plants and a few staple crops [4,7,8,9,10]. In *Arabidopsis thaliana*, a subset of IDD transcription factors (IDD14, IDD15, IDD16) physically interacts with DELLA proteins—key repressors of gibberellin (GA) signaling—to form a functional regulatory module that directly binds to the promoter region of the *FT* (Flowering Locus T) gene, thereby repressing its transcription and modulating flowering time [11,12]. In rice (*Oryza sativa*), *OsIDD10* modulates ammonium acquisition by directly binding to the promoter region of *AMT1.2* and also participates in nitrogen metabolism regulation through its association with the fifth intron of *GDH2* [13], consequently influencing plant architecture and grain yield [14,15]. Moreover, *OsIDD* genes are extensively involved in abscisic acid (ABA)- and gibberellin (GA_3_)-mediated stress responses; for instance, *OsIDD1* and *OsIDD8* positively regulate drought and salt tolerance by transcriptionally activating stress-responsive genes [4]. In maize (*Zea mays*), *ZmIDD14* and *ZmIDD15* are critical regulators of leaf angle formation by modulating cell proliferation and expansion [16], and their expression is significantly induced under heat and osmotic stresses, suggesting functional conservation in abiotic stress adaptation [7]. In wheat and barley, the orthologous protein TaIDD5/HvSDW5 interacts with DELLA proteins (RHT1/SLN1), alleviating DELLA-mediated suppression of GA signaling and thereby promoting cell elongation [17]. Collectively, these findings confirm that IDD proteins execute their regulatory functions primarily through transcriptional activation or repression of target genes and through protein–protein interactions with key components of hormone signaling pathways. Nevertheless, functional characterization of *IDD* genes in major crops remains insufficient compared with that in model plants.

Hexaploid wheat (*Triticum aestivum* L.) is a globally indispensable staple crop that is frequently threatened by biotrophic fungal pathogens, most notably *Blumeria graminis* f. sp. *tritici* (*Bgt*), the causal agent of powdery mildew, and *Puccinia striiformis* f. sp. *tritici* (*Pst*), the causal agent of stripe rust [18,19]. These pathogens severely impair wheat grain yield and quality, posing a major constraint to global wheat production. Despite the well-documented functional significance of *IDD* genes in plant stress adaptation and defense responses, the *TaIDD* gene family in wheat remains insufficiently characterized, and its regulatory roles in mediating defense responses against biotrophic fungal pathogens have not yet been elucidated. To address this critical research gap, we conducted a comprehensive genome-wide identification of the *TaIDD* gene family, systematically analyzing their chromosomal localization, phylogenetic relationships, and conserved domain characteristics. Furthermore, public transcriptomic datasets were used to investigate the expression profiles of *TaIDD* genes under both abiotic and biotic stress conditions, and RT-qPCR was used to validate the expression dynamics of 15 core *TaIDD* genes in response to *Bgt* and *Pst* infection. In this study, we not only delineate the evolutionary conservation and functional diversification of the *TaIDD* gene family, but also identify key candidate genes potentially associated with wheat resistance to biotrophic fungal pathogens, thereby providing a robust theoretical foundation and valuable genetic resources for the molecular breeding of stress-tolerant wheat cultivars.

## 2. Materials and Methods

### 2.1. Genome-Wide Identification of TaIDD Genes

The reference genome sequence, protein sequences, and GFF3 annotation file for common wheat (*Triticum aestivum* cv. Chinese Spring) were obtained from the Ensembl Plants database (https://plants.ensembl.org/index.html, accessed on 13 July 2025). To comprehensively identify *TaIDD* family members, a multiple-strategy approach was implemented. First, protein sequences of known *IDD* genes from *Arabidopsis thaliana* (*AtIDD*) were downloaded from TAIR (https://www.arabidopsis.org/, accessed on 15 July 2025) and then used as queries to perform BLASTP searches (E-value < 1 × 10^−5^) against the wheat proteome using local BLAST 2.12.0+. Concurrently, the hidden Markov model (HMM) profile for the conserved IDD-associated domain was retrieved from the InterPro database (https://www.ebi.ac.uk/interpro/, accessed on 25 May 2026). We performed an integrated search against the InterPro database, restricting the annotation source to PFAM entries only to avoid overlapping predictions. Under “Families, domains, sites & repeats”, only PFAM was selected. Through this refined search, three signature domains of canonical IDD proteins were identified, PF22996 (second C2H2 zinc finger), PF22992 (fourth C2HC zinc finger), and PF22995 (third C2HC zinc finger), with a significance threshold of E-value < 1 × 10^−9^ (Supplementary Table S1). These domains correspond to distinct positions within the conserved zinc finger array and collectively define the IDD family. The candidate sequences retrieved from both homology-based and profile-based searches were subsequently validated using the Conserved Domain Database (CDD) to confirm the presence of definitive IDD domains in the NCBI (https://www.ncbi.nlm.nih.gov/Structure/cdd/wrpsb.cgi, accessed on 18 July 2025) database. Finally, a non-redundant set of genes was designated as the *TaIDD* family and systematically renamed according to their chromosomal order.

### 2.2. Characterization of Physicochemical Properties and Subcellular Localization Prediction

The physicochemical properties of the deduced TaIDD proteins, including the number of amino acids, molecular weight, theoretical isoelectric point (pI), instability index, aliphatic index, and grand average of hydropathicity (GRAVY), were calculated using the Expasy ProtParam tool (https://web.expasy.org/protparam/, accessed on 5 August 2025). Subcellular localization was predicted by integrating the outputs from DeepLoc 2.1 (https://services.healthtech.dtu.dk/services/DeepLoc-2.1/, accessed on 5 August 2025) and WoLF PSORT (https://wolfpsort.hgc.jp/, accessed on 5 August 2025).

### 2.3. Chromosomal Distribution and Phylogenetic and Evolutionary Relationship Analysis of TaIDD Members

Physical chromosomal positions of the identified *TaIDD* genes were mapped using the wheat GFF3 file, and their distribution maps on chromosomes were generated using TBtools II v2.225 [20]. To investigate the evolutionary relationships, full-length protein sequences of *IDD* members from wheat, *Arabidopsis* (*AtIDD*), rice (*OsIDD*), maize (*ZmIDD*), and foxtail millet (*SiIDD*) were aligned using the MUSCLE tool with default parameters: gap open penalty = −2.90, gap extend penalty = 0.00, hydrophobicity multiplier = 1.20, maximum memory = 2048 MB, maximum iterations = 16, and the UPGMA clustering method. A phylogenetic tree was constructed from the aligned sequences using the maximum likelihood method implemented in MEGA v11.0.13 [21], with a bootstrap test of 1000 replicates to assess node confidence. The final tree was visualized and edited using the iTOL website (https://itol.embl.de/upload.cgi, accessed on 6 August 2025).

### 2.4. Analysis of Conserved Motifs, Gene Architecture, and Protein Domains

The conserved protein motifs were discovered using the MEME suite [22] (https://meme-suite.org/meme/tools/meme, accessed on 8 August 2025), with a maximum of 10 motifs. The exon–intron structures of the *TaIDD* genes were visualized based on the genomic and coding sequence alignments, with information extracted from the GFF3 annotation file. An integrated diagram combining the phylogenetic tree, gene structure, and protein domains was generated in TBtools II v2.225.

### 2.5. Gene Duplication, Synteny, and Ka/Ks Analysis of TaIDD Genes

Gene duplication events, including tandem and segmental duplications, were detected using the One Step MCScanX module in TBtools II v2.225 [23], with an e-value threshold of 1 × 10^−10^. The ratios of non-synonymous (Ka) to synonymous (Ks) substitutions (Ka/Ks) for the duplicated gene pairs were calculated to evaluate the selective pressures acting on the *TaIDD* gene family. Furthermore, collinearity analysis among wheat, *Arabidopsis thaliana*, rice, foxtail millet, and maize was performed and visualized using TBtools II v2.225.

### 2.6. Promoter Region Analysis for Cis-Acting Elements

Promoter sequences comprising 2.0 kb of genomic DNA upstream of the predicted translation start site (ATG) for each *TaIDD* gene were extracted. These sequences were subsequently analyzed using the PlantCARE database [24] (https://bioinformatics.psb.ugent.be/webtools/plantcare/html/, accessed on 10 August 2025) to identify putative *cis*-acting regulatory elements. The predicted elements were classified by putative function, including light responsiveness, hormone signaling, stress induction, and developmental regulation. Finally, *cis*-acting elements were visualized using the Gene Structure View module in TBtools II v2.225.

### 2.7. Plant Materials and Stress Treatment

The wheat–*Thinopyrum ponticum* disomic alien substitution line CH10A5 [25] was utilized in this study for expression analysis following biotic stress treatments. CH10A5 exhibits high-level resistance to both powdery mildew (*Blumeria graminis* f. sp. *tritici*, *Bgt*) and stripe rust (*Puccinia striiformi*s f. sp. *tritici*, *Pst*). This line carries a 1J^S^/1D chromosomal substitution, in which chromosome 1D of common wheat is replaced by chromosome 1J^S^ from *Th. ponticum*. Seeds were surface-sterilized, thoroughly rinsed, and germinated in darkness before being grown in a growth chamber (16 h light/8 h dark; 25 °C). At the two-leaf stage, seedlings were independently inoculated with *Puccinia striiformis* f. sp. *tritici* (CYR34) and *Blumeria graminis* f. sp. *tritici* (E09). Leaf tissues were collected at 0, 12, 24, and 48 h post-inoculation (hpi), immediately frozen in liquid nitrogen, and stored at −80 °C for subsequent RNA extraction. Each treatment included three biological replicates.

### 2.8. Expression Profiling of TaIDD Genes

Publicly available transcriptomic sequencing (RNA-Seq) expression studies from various wheat tissues, developmental stages, and under different stress conditions were retrieved from expVIP databases (https://www.wheat-expression.com/, accessed on 10 August 2025) [26]. Specifically, data for drought and heat stress time course in seedlings were obtained from SRP045409 (7 day seedlings age, leaves, 0–6 h, *n* = 2), *Fusarium* head blight (FHB) stress from ERP013829 (flowering stage, spike, 0–48 h, *n* = 6), stripe rust and powdery mildew stress infection time course from SRP041017 (7 day seedlings, leaves, 0–72 h, *n* = 3), and *Zymoseptoria tritic*i stress from ERP009837 (17 day seedlings, leaves, 0–21 days, *n* = 3). The expression levels of *TaIDD* genes were quantified as transcripts per million (TPM) using Salmon v1.4.0 based on the IWGSC RefSeq v2.1 annotation; TPM values were normalized and analyzed separately for each dataset and transformed with log2(TPM+1) for visualization. Differential expression was analyzed with DESeq2 (|log2FC| > 1, adjusted *p*-value < 0.05, Benjamini–Hochberg correction); for time-course data, ANOVA with Tukey’s post hoc test was applied across time points. A heatmap was subsequently generated using TBtools II v2.225 to visualize the spatiotemporal expression patterns across these diverse conditions.

### 2.9. RT-qPCR Analysis

RT-qPCR was performed for experimental validation. Total RNA was extracted using the UNIQ-10 Column Trizol Total RNA Isolation Kit (B51131), and cDNA was synthesized using the MightyScript Plus First Strand cDNA Synthesis Master Mix (gDNA digester) (B639252), followed by RT-qPCR with the 2X HyperMB Universal SYBR Green qPCR Master Mix (B690016). All reagents were purchased from Sangon Biotech (Shanghai, China) Co., Ltd. To characterize the overall transcriptional activity of each *TaIDD* gene locus across the three subgenomes, gene-specific primers were designed using Primer-BLAST (NCBI) based on conserved regions within the coding sequence and 3′-UTR that are shared among the A, B, and D homoeologs. Consequently, the primers amplify all three homoeologous copies simultaneously rather than being subgenome-specific. This approach was intentionally chosen because (i) the IDD domain and its flanking regions are highly conserved among the three homoeologs, making subgenome-specific primer design technically challenging and potentially prone to amplification bias; and (ii) our primary objective was to quantify the combined expression level of each *TaIDD* gene copy number rather than resolving subgenome-specific contributions. All primers were validated by standard curve analysis (90% < efficiency < 110%, R^2^ > 0.98), with specificity confirmed by melting curve analysis (single peak) and 1.5% agarose gel electrophoresis (single band of expected size), ensuring that the amplicons represent the intended conserved targets without non-specific amplification. Primer sequences are listed in Table S6.

RT-qPCR was performed on the QuantStudio 6 Flex Real-Time PCR System (Thermo Fisher Scientific, Waltham, MA, USA) with three biological replicates per treatment, each with three technical replicates. *TaActin* was used as the internal reference gene. Relative expression levels were normalized against TaActin and quantified using the 2^−ΔΔCT^ method. Results are expressed as mean ± SEM of three biological replicates. Statistical significance was assessed via one-way ANOVA with Tukey’s test.

### 2.10. Subcellular Localization

To determine the subcellular localization of TaIDD, its coding sequence (without the stop codon) was inserted into the *pCAMBIA1302* vector to create a C-terminal GFP fusion. This construct was introduced into *Agrobacterium* strain GV3101 and transiently expressed in *N. benthamiana* leaves via agroinfiltration. An empty *pCAMBIA1302-GFP* vector was used as a negative control. Fluorescence signals were captured using a spinning disk confocal laser-scanning microscope (UltraView VoX, PerkinElmer, Waltham, MA, USA). The excitation wavelength was set to 488 nm (UltraView VoX), and emissions were recorded through a 525/50 nm band-pass filter. For each independent transformation, at least 10 cells per field of view were examined across multiple visual fields, and representative images displaying consistent localization patterns were captured. All experiments were conducted with three biological replicates.

## 3. Results

### 3.1. Identification and Basic Physicochemical Properties of TaIDD Members

Through genome-wide screening and validation using BLASTP, HMMER, and conserved domain analysis, a total of 41 non-redundant IDD transcription factors were identified in common wheat (*Triticum aestivum*) and designated *TaIDD1* to *TaIDD41* (Supplementary Table S2). Biochemical characterization revealed considerable variation in physicochemical properties among TaIDD proteins. Amino acids (aa) lengths ranged from 405 aa (TaIDD24) to 845 aa (TaIDD7), with molecular weights (MWs) ranging from 42.92 to 92.01 kDa and theoretical isoelectric points (pI) varying from acidic (5.41, TaIDD4) to basic (9.66, TaIDD27), indicating diverse electrostatic characteristics. Instability indices suggested that several members may be unstable in vitro. All TaIDD proteins were predicted to be hydrophilic, as indicated by a consistently negative grand average of the hydropathicity (GRAVY) values (−0.754 to −0.435). Subcellular localization prediction supported nuclear localization for all 41 TaIDD proteins, providing a structural and bioinformatic basis for future functional studies.

### 3.2. Chromosomal Localization and Phylogenetic Analysis of the IDD Gene Family in Wheat

**Figure 1:**
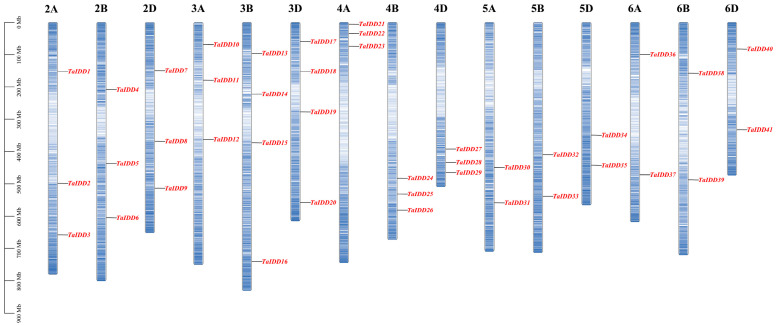
Chromosome distribution of *TaIDD* members in the wheat genome.

Chromosomal localization analysis revealed that the *TaIDD* gene family is unevenly distributed across the 21 chromosomes of hexaploid wheat (Figure 1). All *TaIDD* genes were mapped to 15 chromosomes belonging to homoeologous groups 2, 3, 4, 5, and 6, with no members detected on homoeologous groups 1 and 7. Specifically, the highest gene densities were observed on chromosome 3B (4 genes). This uneven distribution pattern may potentially reflect the complex evolutionary history of hexaploid wheat, including possible contributions from polyploidization events. However, the specific mechanisms underlying *TaIDD* family expansion remain to be elucidated.

**Figure 2:**
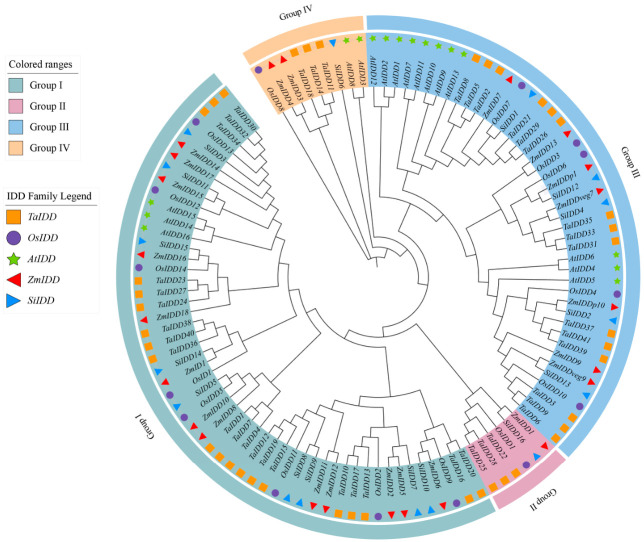
Phylogenetic analysis of IDD proteins from *Arabidopsis thaliana* (At), *Oryza sativa* (Os), *Zea mays* (Zm), *Setaria italica* (Si), and *Triticum aestivum* (Ta). The phylogenetic tree was inferred using the maximum likelihood (ML) method with MEGA 11 software (version 11.0.13).

To elucidate the evolutionary relationships among *TaIDD* genes and their orthologs across plant species, a maximum-likelihood phylogenetic tree was constructed using full-length IDD protein sequences from wheat (TaIDD), rice (OsIDD), *Arabidopsis thaliana* (AtIDD), maize (ZmIDD), and *Setaria italica* (SiIDD) (Figure 2, Supplementary Table S3). The *IDD* gene family was clearly divided into four phylogenetic groups (Group I–IV). Group I, the largest group, contained the majority of *TaIDD* genes alongside orthologs from all five examined species, indicating a conserved evolutionary origin spanning both monocot grasses and dicot plants. In contrast, Group II formed a smaller, monocot-specific clade comprising a subset of *TaIDD* genes and their close orthologs in rice and maize, suggesting potential grass lineage-specific functional divergence. Finally, Groups III and IV contained the remaining *TaIDD* genes, which clustered with other grass orthologs, reflecting lineage-specific expansion within the Poaceae. This conserved grouping suggests that major functional divergence within the *IDD* family predated the speciation of these lineages. Notably, TaIDD proteins were distributed across all groups (with a predominance in Groups I and II) and consistently showed closer evolutionary ties to grass orthologs (rice, maize) than to *Arabidopsis*, highlighting a shared evolutionary trajectory among cereal crops.

### 3.3. Comparative Analysis of Gene Structure and Protein Features of TaIDD Family Members

**Figure 3:**
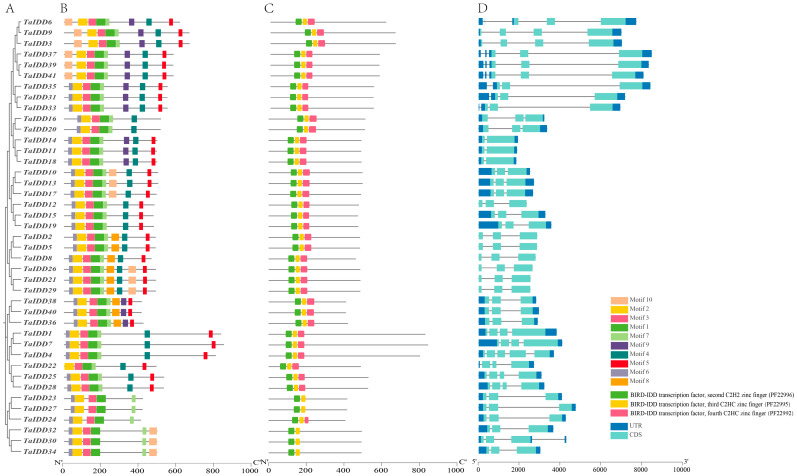
Comprehensive analysis of *TaIDD* gene family members. (**A**) Phylogenetic tree showing evolutionary relationships. (**B**) MEME-based visualization of conserved motifs, with colored boxes representing specific conserved amino acid sequences. (**C**) Conserved domain architecture inferred from InterPro analysis, with color-coded boxes indicating phylogenetically conserved functional modules. (**D**) Gene structure analysis depicting exon–intron organization of *TaIDD* members.

To gain deeper insight into the structural evolution and functional diversification of the *TaIDD* family, a comprehensive comparative analysis of gene structure and conserved protein features was conducted and is visually summarized in Figure 3. First, an evolutionary tree was constructed for the *TaIDD* members using MEGA 11 software, with these categorized into four groups (Figure 3A). Conserved motif analysis revealed that the *TaIDD* gene family members shared similar motif compositions, with 10 motifs distributed in a relatively conserved manner across most *TaIDD* members (Figure 3B), suggesting that these motifs may play important roles in TaIDD protein function. Notably, some *TaIDD* members exhibited variations in motif composition. For example, *TaIDD23*, *TaIDD24*, and *TaIDD27* uniquely lacked multiple conserved motifs, and several members (*TaIDD6*, *TaIDD10*, *TaIDD32*) lacked rearrangement of specific motifs, suggesting potential isoform-specific functional modifications that warrant further experimental validation. Furthermore, all *TaIDD* members harbored the typical conserved IDD domain, a hallmark feature of *IDD* transcription factors containing a C2H2 zinc finger motif, which confirms their functional identity as IDD proteins involved in transcriptional regulation (Figure 3C). Gene structure analysis revealed substantial variation in exon–intron organization among *TaIDD* members (Figure 3D). Despite a high degree of conservation in motif composition, protein domains, and gene structure, some variations were observed among *TaIDD* members. For example, the lengths of the CDS regions and the positions of some motifs may differ among different members, which may be related to the functional diversification of the *TaIDD* gene family. Overall, the structural analysis of the *TaIDD* gene family members shows a high degree of conservation in core domains while exhibiting flexibility in gene architecture, providing important insights into the functional conservation and evolution of this gene family.

### 3.4. Evolutionary Dynamics of the TaIDD Gene Family Revealed Through Synteny and Selective Pressure Analyses

**Figure 4:**
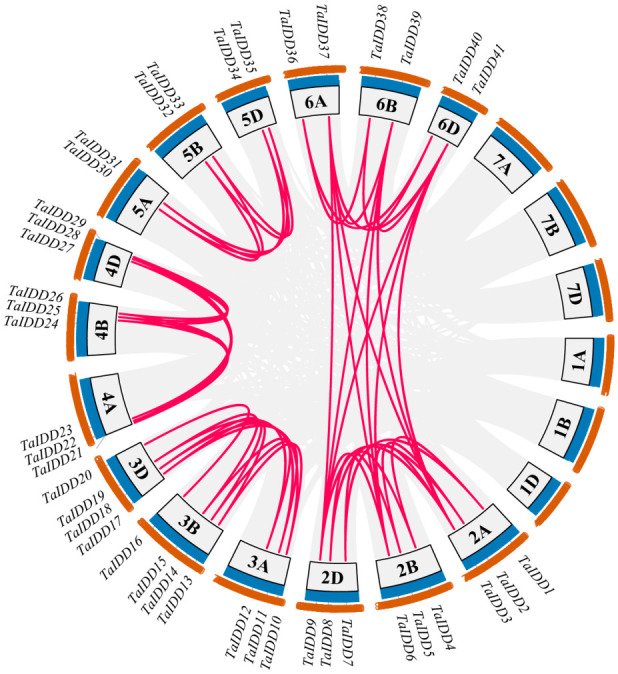
Genomic collinearity analysis of *TaIDD* gene family members. Red lines indicate gene pairs with collinearity relationships, numbers within the boxes represent chromosomes, and light blue boxes denote gene density.

Intra-species syntenic analysis of the *TaIDD* gene family in wheat revealed that *TaIDD* genes were unevenly distributed across 15 wheat chromosomes, with duplicated gene pairs predominantly located on homoeologous groups 2–6 (Figure 4). Segmental duplication events were identified as the primary driver of *TaIDD* family expansion, with 49 segmentally duplicated gene pairs detected, reflecting conserved synteny among the A, B, and D subgenomes of hexaploid wheat (Supplementary Table S4). Calculation of non-synonymous (Ka) and synonymous (Ks) substitution rates for all duplicated *TaIDD* pairs exhibited Ka/Ks values ranging from 0.0308 to 0.6406 (average ~0.25), all significantly less than 1, suggesting that duplicated *TaIDD* genes may have undergone strong purifying selection to maintain functional integrity and conservation during wheat evolution.

**Figure 5:**
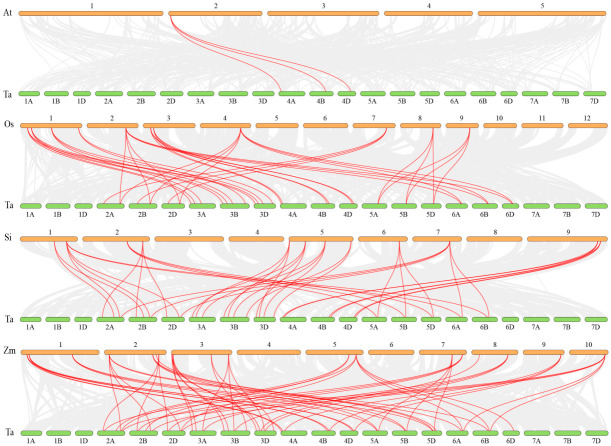
Interspecific collinearity analysis of *IDD* gene family members. Collinearity analysis between wheat and *Arabidopsis thaliana*, rice, maize, and foxtail millet. Species abbreviations: Ta, *Triticum aestivum*; At, *Arabidopsis thaliana*; Os, *Oryza sativa*; Si, *Setaria italica*; Zm, *Zea mays*.

Interspecific collinearity analysis between wheat and four representative species (*Arabidopsis thaliana*, *Oryza sativa*, *Setaria italica*, *Zea mays*) further illustrated the evolutionary conservation of the *IDD* gene family. Limited collinear *IDD* gene pairs were detected between wheat and *A. thaliana* (a eudicot), whereas extensive, highly conserved collinear relationships were observed between wheat and the three grass species (Figure 5). This pattern highlights the stable synteny of *IDD* genes within the Poaceae family and the substantial disruption of collinearity following the divergence of monocots and eudicots, suggesting that the *IDD* gene family may have arisen before the diversification of major grass lineages and may have maintained conserved genomic positions throughout *Poaceae* evolution.

### 3.5. Analysis of Stress- and Hormone-Responsive Cis-Acting Elements

**Figure 6:**
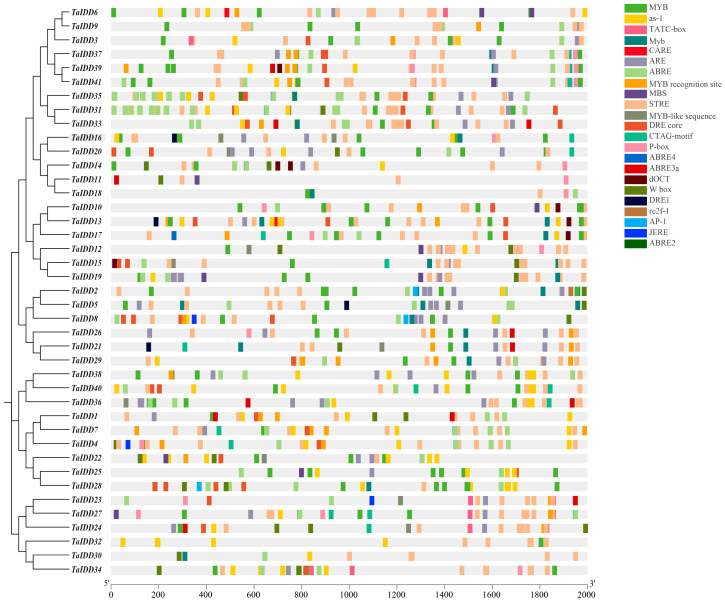
*Cis*-acting regulatory elements in the promoter regions of *TaIDD* genes.

To elucidate the potential regulatory mechanisms underlying the growth and stress-responsive functions of *TaIDD* genes, we analyzed the distribution of cis-acting elements in the 2000 bp promoter regions. A total of 24 distinct *cis*-elements were identified and classified into three major functional categories: hormone response elements, stress response elements, and developmental regulation elements (Figure 6, Supplementary Table S5). Hormone-related elements were abundantly represented, including ABA-responsive elements (ABRE/ABRE2/ABRE3a/ABRE4), JA-responsive elements (JERE), and GA-responsive elements (TATC-box), suggesting that *TaIDD* genes may potentially be regulated by major phytohormone signaling pathways. Stress-responsive elements, such as drought-responsive elements (DRE core, DRE1), broad-spectrum stress elements (STRE), low-oxygen responsive elements (ARE), and cold-responsive elements (CARE), were also prevalent, implying a possible involvement of *TaIDD* genes in abiotic stress adaptation. Additionally, elements associated with developmental processes, including MYB/MBS/MYB-like sequences (secondary metabolism and stomatal development) and dOCT (developmental transition), were detected (Figure 6). The heterogeneous distribution of these elements across *TaIDD* members suggests potential functional divergence, with individual genes possibly participating in distinct regulatory networks that may coordinate wheat growth, development, and stress resilience.

### 3.6. Response Patterns of TaIDD Genes Under Abiotic and Biotic Stresses

**Figure 7:**
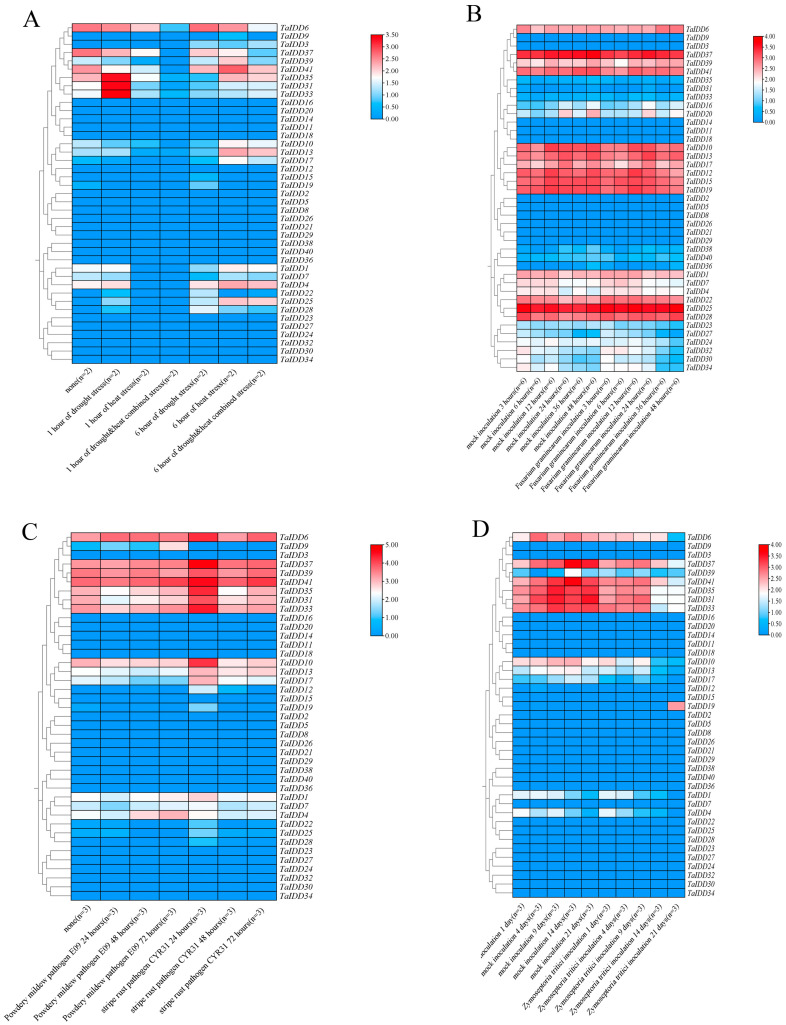
Expression patterns of *TaIDD* genes in response to abiotic and biotic stresses. (**A**) drought and heat stresses. (**B**) *Fusarium* head blight (FHB) stress. (**C**) stripe rust and powdery mildew stresses. (**D**) *Zymoseptoria tritici* stress.

Expression patterns of *TaIDD* genes under multiple abiotic and biotic stresses were analyzed based on public transcriptome data. Under drought stress, most *TaIDD* genes remained at low expression levels, while a subset, including *TaIDD31*, *TaIDD33*, and *TaIDD35,* exhibited significant upregulation at early stress time points (Figure 7A), suggesting their potential involvement in rapid responses to drought stress.

In response to biotic stress, distinct expression patterns were observed across four different pathogens. Under *Fusarium graminearum* inoculation, *TaIDD37*, *TaIDD39*, *TaIDD10*, *TaIDD13*, *TaIDD17*, and *TaIDD25* exhibited strong and sustained upregulation throughout the infection process (3–48 h) (Figure 7B), implying a possible association with defense responses against *Fusarium* head blight (FHB). For powdery mildew and stripe rust stresses, *TaIDD17*, *TaIDD37*, *TaIDD39*, and *TaIDD41* were specifically induced at 24–48 h post-inoculation (Figure 7C), displaying pathogen-responsive expression patterns. In contrast, under *Zymoseptoria tritici* inoculation, only *TaIDD31*, *TaIDD33*, *TaIDD35*, *TaIDD37*, and *TaIDD41* exhibited moderate upregulation at early time points, while most other *TaIDD* genes maintained low expression levels (Figure 7D).

Notably, *TaIDD39*, *TaIDD41*, and *TaIDD35* were consistently upregulated under multiple biotic stresses, suggesting that they may be candidate genes potentially associated with stress-responsive pathways related to disease resistance. In addition, genes such as *TaIDD10* and *TaIDD19* exhibited pathogen-specific induction, indicating possible specialized roles in defense against particular pathogens. These results reveal that the *TaIDD* gene family displays diverse and stress-specific expression patterns, suggesting potential functional differentiation in mediating wheat responses to both abiotic and biotic stresses.

### 3.7. RT-qPCR Validation of TaIDD Gene Expression Under Powdery Mildew and Stripe Rust Stress

To analyze transcriptomic data and examine changes in key *TaIDD* gene expression during wheat defense, 15 candidate genes were tested by means of RT-qPCR after infection with the pathogens *Blumeria graminis* f. sp. *tritic*i (*Bgt*, E09) and *Puccinia striiformis* f. sp. *tritici* (*Pst*, CYR34).

**Figure 8:**
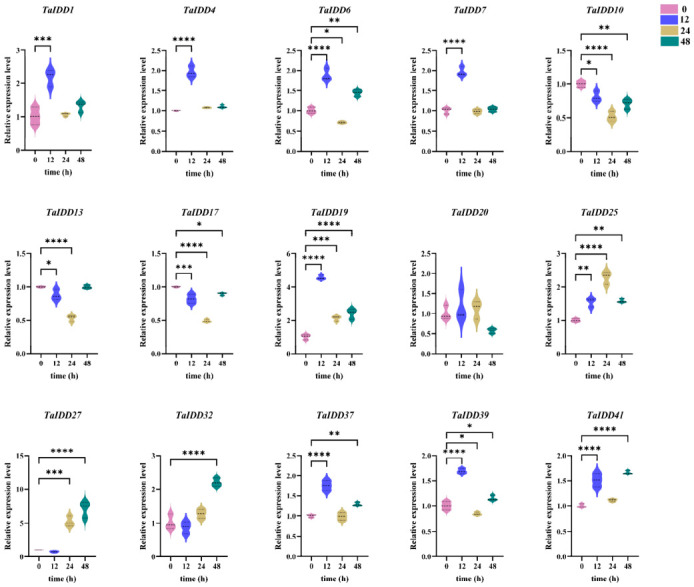
RT-qPCR analysis of *TaIDD* gene expression in response to powdery mildew (E09). Relative expression levels of 15 *TaIDD* genes at 0, 12, 24, and 48 h post-inoculation (hpi) with *Blumeria graminis* f. sp. *tritici* (*Bgt*) isolate E09. Purple: 0 hpi; blue: 12 hpi; yellow: 24 hpi; green: 48 hpi. Significance levels: * *p* < 0.05; ** *p* < 0.01; *** *p* < 0.001; **** *p* < 0.0001. Data are presented as the mean ± SEM of three biological replicates (*n* = 3).

Under E09 inoculation, the 15 *TaIDD* genes exhibited distinct temporal expression trends (Figure 8). Specifically, at 12 hpi, a subset—including *TaIDD1*, *TaIDD4*, *TaIDD6*, *TaIDD7*, *TaIDD19*, *TaIDD25*, *TaIDD37*, *TaIDD39*, and *TaIDD4*1—was rapidly and significantly upregulated. Notably, *TaIDD19* exhibited the strongest induction, with more than a 4-fold increase at 12 hpi relative to 0 h, suggesting its possible involvement in early *Bgt* recognition and response. Moreover, *TaIDD27* exhibited the strongest induction, with a more than 5-fold increase at 24 hpi. By 48 hpi, while most induced gene expression subsided, *TaIDD19*, *TaIDD25*, *TaIDD27*, *TaIDD32*, *TaIDD39*, and *TaIDD41* maintained moderately elevated levels compared to the control, implying their potential continued association with later defense responses against powdery mildew.

**Figure 9:**
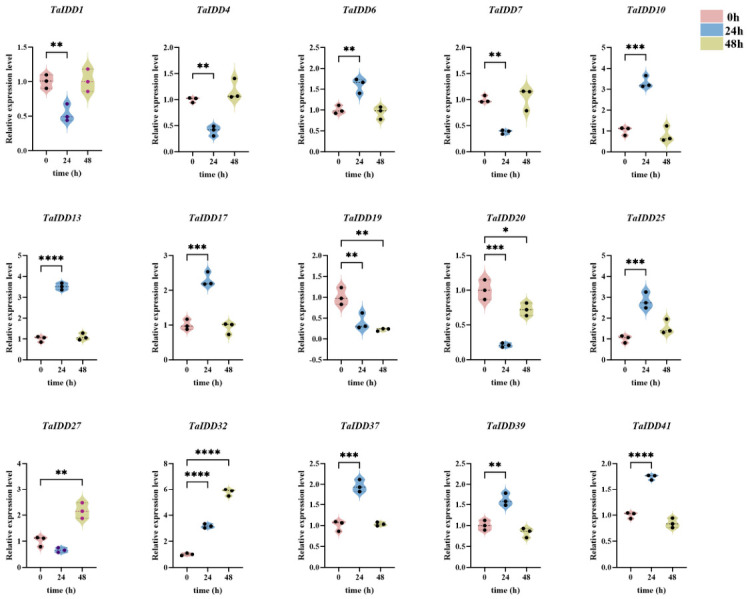
RT-qPCR analysis of *TaIDD* gene expression in response to stripe rust (CYR34). Relative expression levels of 15 *TaIDD* genes at 0, 24, and 48 h post-inoculation (hpi) with *Puccinia striiformis* f. sp. *tritici* (*Pst*) isolate CYR34. Purple: 0 h; blue: 24 h; yellow: 48 h. Significance levels: * *p* < 0.05; ** *p* < 0.01; *** *p* < 0.001; **** *p* < 0.0001. Data are presented as the mean ± SEM of three biological replicates (*n* = 3).

In response to CYR34 infection, the expression patterns of the *TaIDD* genes exhibited differential magnitudes (Figure 9). The most pronounced induction was observed for *TaIDD6*, *TaIDD10*, *TaIDD13*, *TaIDD17*, *TaIDD25*, *TaIDD32*, *TaIDD37*, *TaIDD39*, and *TaIDD41*, all of which were robustly upregulated as early as 24 hpi. Specifically, *TaIDD13* exhibited the highest expression levels, with approximately 3- to 4-fold upregulation at 24 hpi, suggesting its possible association with resistance responses to stripe rust. At 48 hpi, genes such as *TaIDD27* and *TaIDD32* remained significantly induced, while *TaIDD6*, *TaIDD10*, *TaIDD13*, *TaIDD17*, *TaIDD37*, *TaIDD39*, and *TaIDD41* returned to near-baseline levels, reflecting the precise temporal regulation of *TaIDD* genes during the incompatible interaction with *Pst*.

Collectively, the RT-qPCR results validated the transcriptome data. The selected *TaIDD* genes are widely involved in wheat responses to both powdery mildew and stripe rust stresses. A core set of genes—including *TaIDD6*, *TaIDD25*, *TaIDD27*, *TaIDD37*, *TaIDD39* and *TaIDD41*—exhibited induction in response to both pathogens, suggesting that they may represent candidate genes potentially associated with broad-spectrum stress-responsive pathways. These genes exhibited rapid and significant upregulation at the early infection stage. This finding suggests that they may act as conserved key regulators in broad-spectrum disease resistance. Furthermore, genes such as *TaIDD1*, *TaIDD4*, *TaIDD7*, *TaIDD10*, *TaIDD13*, *TaIDD17,* and *TaIDD19* exhibited pathogen-specific expression patterns, indicating possible functional specialization in the regulatory networks underlying wheat interactions with distinct biotrophic pathogens. These findings provide a foundation for future functional dissection of the *TaIDD* gene family, with their potential application in molecular breeding for durable disease resistance requiring further genetic validation.

### 3.8. Subcellular Localization of TaIDD39 Protein

**Figure 10:**
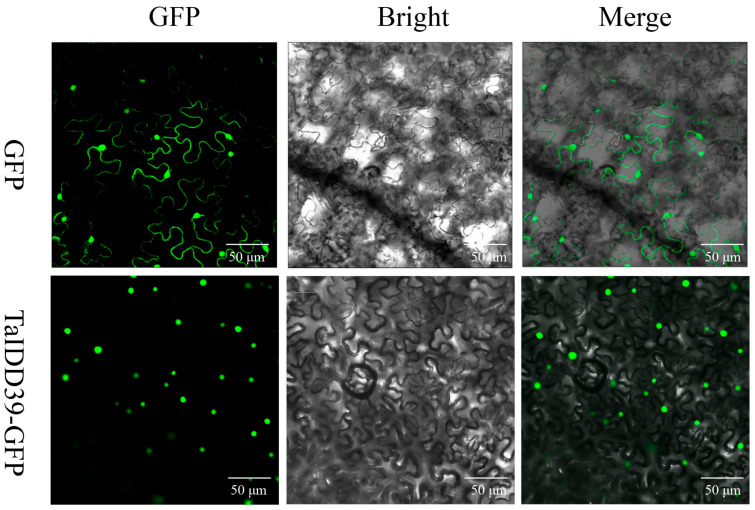
Subcellular localization of the TaIDD39 protein. Scale bars correspond to 50 μm.

Given the significant induction of *TaIDD39* in response to abiotic and biotic challenges, we hypothesized its potential role in mediating stress resilience in wheat. To investigate the subcellular distribution of TaIDD39 as a putative transcription factor, we performed transient expression assays using a TaIDD39-GFP fusion construct in *Nicotiana benthamiana*. The fluorescence signal was predominantly detected in the nucleus, substantiating the hypothesis that TaIDD39 functions as a nuclear-localized transcription factor (Figure 10). This result is consistent with the presence of a predicted nuclear localization signal in the IDD domain and the expected subcellular distribution of transcriptional regulators.

## 4. Discussion

### 4.1. Evolutionary Conservation and Functional Divergence of the TaIDD Gene Family

In this study, 41 *TaIDD* transcription factors were systematically identified at the whole-genome level in hexaploid wheat. Through phylogenetic analysis, these TaIDD proteins were classified into four distinct groups, which exhibited closer homology with IDD proteins from gramineous crops, including rice, maize, and foxtail millet, than with those from *Arabidopsis thaliana*. These results suggest that the *IDD* gene family underwent lineage-specific expansion after the divergence of monocots and dicots and remains highly conserved in the grass family (*Poaceae*) [27,28]. Chromosomal localization revealed that *TaIDD* genes were unevenly distributed on homoeologous groups 2, 3, 4, 5, and 6, with no distribution on groups 1 and 7, suggesting that gene retention and loss occurred during wheat polyploidization [29].

Analysis of gene structure and conserved motifs demonstrated that *TaIDD* members within the same phylogenetic group shared similar exon–intron arrangements and motif compositions, whereas clear structural variations were observed among different groups. Some *TaIDD* genes exhibited motif deletion or rearrangement, suggesting possible functional divergence. All TaIDD proteins harbored the typical IDD domain and C2H2 zinc finger motif and were predicted to localize in the nucleus, consistent with their molecular function as transcription factors [4,16,30].

Intraspecies synteny analysis revealed that segmental duplication contributed predominantly to the expansion of the *TaIDD* family [31]. All duplicated gene pairs exhibited Ka/Ks ratios significantly less than 1, suggesting that the *TaIDD* gene family may have undergone strong purifying selection to maintain functional stability during evolution, consistent with the evolutionary characteristics of stress-responsive gene families in wheat [32,33]. Interspecies collinearity analysis highlighted highly conserved syntenic relationships between wheat and other gramineous species but limited collinearity between wheat and *Arabidopsis*, supporting the ancient origin and high evolutionary conservation of *IDD* genes in *Poaceae* [2,34].

### 4.2. Cis-Acting Architecture and Hormonal Cross-Talk Reveal the Versatile Regulatory Potential of TaIDD Genes

The comprehensive analysis of cis-acting elements in *TaIDD* promoter regions reveals a sophisticated regulatory architecture that integrates hormonal signaling with stress-responsive pathways. The abundance of ABA-, JA-, and GA-responsive elements suggests that *TaIDD* genes may potentially function as central nodes in phytohormone cross-talk networks, possibly coordinating the transition between defense and stress tolerance strategies [17]. ABA and JA are pivotal mediators of abiotic and biotic stress responses, respectively, and their co-occurrence in *TaIDD* promoters indicates the possibility that these transcription factors may prioritize resource allocation between growth, stress tolerance, and defense [35,36]. This hormonal integration could be crucial for wheat, as simultaneous exposure to drought, heat, and pathogen pressure may require the precise coordination of competing physiological demands [37]. Notably, the co-occurrence of drought-responsive elements (DRE core) with broad-spectrum stress elements (STREs) and developmental regulators (MYB/MBSs) implies that *TaIDD* genes may potentially mediate the interface between abiotic stress adaptation and developmental transitions, such as the shift from vegetative growth to reproductive phase under environmental constraints [38]. The presence of these developmental elements alongside stress-responsive motifs suggests that *TaIDD* genes may potentially bridge growth-defense trade-offs, a fundamental challenge in crop improvement.

The heterogeneous distribution of these elements across family members suggests that individual *TaIDD* genes have undergone promoter diversification, potentially enabling the family as a whole to respond to a wide spectrum of stimuli while maintaining specificity in regulatory outputs [39]. This modular organization implies possible functional partitioning, where specific *TaIDD* genes may specialize in particular stress responses while others maintain developmental functions. Such regulatory flexibility could be particularly advantageous in polyploid wheat, where duplicated genes can potentially acquire distinct expression patterns through cis-element variation, thereby expanding the functional repertoire of the family. This observation offers potential opportunities for targeted manipulation: identifying and engineering promoter variants with enhanced stress-responsive element compositions may potentially decouple defense activation from yield penalties, a major bottleneck in resistance breeding. This regulatory complexity is particularly relevant in the context of wheat improvement, where the simultaneous optimization of yield potential and stress resilience remains a paramount challenge. The identification of *TaIDD* genes enriched for specific hormone-responsive elements provides candidate molecular targets for breeding strategies aimed at fine-tuning hormone sensitivity to enhance stress tolerance without compromising agronomic performance [40]. For instance, genes harboring both ABA- and JA-responsive elements could be prioritized as candidates for enhancing broad-spectrum stress resistance, while those with predominantly developmental motifs may be targeted for yield-related traits, potentially enabling the development of allele-specific markers and promoter-editing strategies for climate-resilient wheat varieties.

### 4.3. Expression Characteristics and Functional Diversification of TaIDD Genes Under Multiple Stresses

Transcriptomic profiling demonstrated that most *TaIDD* genes exhibited low expression under drought stress. However, *TaIDD31*, *TaIDD33*, and *TaIDD35* were rapidly and strongly induced at 1 h, suggesting their potential involvement as early-responsive regulators in abiotic stress adaptation [41]. Under biotic stress, *TaIDD* genes displayed pathogen-specific expression patterns. *TaIDD20* and *TaIDD16* were specifically induced by Fusarium graminearum, suggesting possible specialized roles in response to FHB. In contrast, *TaIDD35*, *TaIDD39*, and *TaIDD41* were consistently upregulated by powdery mildew, stripe rust, *F. graminearum*, and *Zymoseptoria tritici*, suggesting that they may represent candidate genes potentially associated with broad-spectrum stress-responsive pathways related to disease resistance [ [42,43,44]. RT-qPCR verification further confirmed that *TaIDD37*, *TaIDD39*, *TaIDD41*, *TaIDD25,* and *TaIDD6* were significantly induced at early stages during *Bgt* and *Pst* infection, supporting their potential association with defense responses against biotrophic fungal pathogens. The pathogen-specific expression patterns of *TaIDD1* and *TaIDD13* suggest that the *TaIDD* family may have diverged to fine-tune defense responses against different pathogens, possibly by integrating the SA and JA signaling pathways [45]. However, we emphasize that transcriptional induction alone does not demonstrate functional involvement in resistance pathways; direct experimental validation, including pathogen growth assays, mutant analysis, and downstream defense marker studies, is essential to establish any causal roles.

Because *TaIDD* genes are not located on homoeologous group 1 chromosomes, the 1J^S^/1D substitution does not directly overlap with the genomic regions harboring *TaIDD* loci. Nevertheless, we recognize that the use of the alien substitution line CH10A5 may limit the direct extrapolation of our expression data to common wheat cultivars. The 1J^S^/1D substitution, while not directly affecting *TaIDD* gene loci, could influence the broader regulatory environment through chromatin-level or trans-acting effects. To address this concern, we utilized the public transcriptome dataset SRP041017, which was generated from common wheat in our laboratory, as an independent in-house validation control to specifically examine whether the introgression of the alien 1J^S^ chromosome perturbs *IDD* gene expression in the common wheat background. The biotic stress-induced expression patterns reported here should be considered representative of the CH10A5 genotype specifically, and follow-up studies in elite common wheat varieties are warranted to validate these findings.

The distinct expression patterns of *TaIDD* genes under abiotic versus biotic stresses suggest a sophisticated regulatory system that may discriminate between different environmental challenges. *TaIDD37*, *TaIDD39,* and *TaIDD41* are rapidly upregulated under combined drought stress, suggesting potential roles in early stress sensing or signal amplification as candidate ‘trigger’ genes that may initiate but not necessarily sustain responses [36]. Conversely, *TaIDD39* and *TaIDD41* are upregulated for longer periods during *F. graminearum* infection, indicating possible associations with defense responses, potentially through the direct regulation of pathogenesis-related genes or by strengthening the cell wall [46]. The consistent upregulation of *TaIDD37*, *TaIDD39*, and *TaIDD41* across multiple biotrophic pathogens represents an intriguing expression pattern that warrants further functional investigation. While their strong induction at 24 hpi aligns temporally with the shift from pathogen recognition to defense, suggesting that they may potentially contribute to resistance regulation, we caution that this correlation does not establish causation [47]. Their co-expression with *Bgt* and *Pst* infection suggests that they are responsive to pathogen challenge; however, whether they function as positive regulators of the SA or ROS signaling pathways remains to be elucidated through genetic manipulation studies [42,48]. In this study, TaIDD39 was found to be localized to the nucleus. This subcellular distribution is in line with its predicted function as a transcription factor and lends mechanistic evidence to the hypothesis of its regulatory action, as nuclear access is a prerequisite for controlling defense gene transcription. Furthermore, the varied expression characteristics of *TaIDD* family members identify valuable candidates for subsequent functional research on their contributions to wheat abiotic stress tolerance and disease resistance [49].

### 4.4. Limitations of This Study

We acknowledge several limitations of the present study. First, the expression analysis was conducted at 12, 24, and 48 hpi, which captures the transition from pathogen recognition to activated defense responses but does not include earlier time points (e.g., 0–6 hpi) that would capture the immediate transcriptional responses during the earliest stages of pathogen recognition and PAMP-triggered immunity. Future studies incorporating 6 hpi and earlier time points will be essential to fully resolve the temporal dynamics of *TaIDD* gene induction. Second, the biotic stress expression data were generated using the alien substitution line CH10A5, and although the experimental rationale for this choice is sound, validation in common wheat cultivars is needed to confirm the generalizability of these expression patterns. Third, the RT-qPCR primers used in this study amplified all three homoeologous copies simultaneously; subgenome-resolved expression analysis using allele-specific or subgenome-specific approaches will provide a more nuanced understanding of homoeolog-specific contributions to stress responses.

### 4.5. Significance and Perspectives

In this study, we provide a systematic characterization of the genomic characteristics, evolutionary patterns, structural features, and stress-responsive expression profiles of the wheat *TaIDD* gene family. The *TaIDD* family is evolutionarily conserved, with expression patterns suggestive of potential functional divergence among members in response to abiotic and biotic stresses. *TaIDD37*, *TaIDD39*, and *TaIDD41* were identified as candidate genes exhibiting transcriptional responsiveness to multiple stresses, potentially associated with stress-responsive pathways related to disease resistance. However, we emphasize that these genes require functional validation before their utility in breeding applications can be assessed. Future studies incorporating gene editing, protein–protein interaction assays, and transcriptome sequencing may help elucidate the molecular mechanisms of *TaIDD* genes in wheat defense against fungal pathogens, providing a reference framework for potential genetic improvement of stress-resistant wheat cultivars [50,51].

## 5. Conclusions

In conclusion, we systematically identified 41 *TaIDD* genes in common wheat and characterized their phylogenetic relationships, gene structures, chromosomal locations, synteny, and *cis*-acting elements. Expression analyses revealed that *TaIDD* genes exhibit diverse transcriptional responses to various abiotic and biotic stresses, with *TaIDD13, TaIDD19*, *TaIDD27*, *TaIDD37*, *TaIDD39*, and *TaIDD41* exhibiting notable induction under powdery mildew and stripe rust infection. These findings suggest that the *TaIDD* gene family may possess diverse roles in stress-responsive pathways. Our findings reveal valuable candidate genes and provide a theoretical basis for the molecular breeding of disease-resistant wheat. In addition, this study provides a foundational resource and highlights candidate genes for future functional investigations aimed at elucidating the potential roles of *TaIDD* family members in wheat stress adaptation and disease resistance.

## Data Availability

The original contributions presented in this study are included in the article. Further inquiries can be directed to the corresponding author.

## Supplementary Materials

The following supporting information can be downloaded at https://www.mdpi.com/article/10.3390/biology15120904/s1. Table S1: *TaIDD* identification via the InterPro database. Table S2: Protein sequence information of *TaIDD* genes identified in this study; Table S3: Accession numbers of *AtIDDs*, *OsIDDs*, *ZmIDDs,* and *SiIDDs*; Table S4: Ratio of Ka/Ks and distribution of duplicate *TaIDD* genes; Table S5: Characteristics of cis-acting regulatory elements in the promoter region of the identified *TaIDD* genes; Table S6: Primer sequences used for *TaIDD* genes.

## Abbreviations

The following abbreviations are used in this manuscript:

RT-qPCR	Reverse transcription-quantitative polymerase chain reaction
pI	Isoelectric points
GRAVY	Grand average of hydropathy
ABA	Abscisic acid
FHB	*Fusarium* head blight
*Bgt*	*Blumeria graminis* f. sp. *tritici*
*Pst*	*Puccinia striiformis* f. sp. *tritici*
